# Involvement of anti-tumor *miR-124-3p* and its targets in the pathogenesis of pancreatic ductal adenocarcinoma: direct regulation of *ITGA3* and *ITGB1* by *miR-124-3p*

**DOI:** 10.18632/oncotarget.25599

**Published:** 2018-06-22

**Authors:** Tetsuya Idichi, Naohiko Seki, Hiroshi Kurahara, Haruhi Fukuhisa, Hiroko Toda, Masataka Shimonosono, Yasutaka Yamada, Takayuki Arai, Yoshiaki Kita, Yuko Kijima, Yuko Mataki, Kosei Maemura, Shoji Natsugoe

**Affiliations:** ^1^ Department of Digestive Surgery, Breast and Thyroid Surgery, Graduate School of Medical Sciences, Kagoshima University, Kagoshima, Japan; ^2^ Department of Functional Genomics, Chiba University Graduate School of Medicine, Chiba, Japan

**Keywords:** microRNA, miR-124-3p, ITGA3, ITGB1, pancreatic ductal adenocarcinoma

## Abstract

MicroRNAs (miRNAs) are unique in that a single miRNA molecule regulates a vast number of RNA transcripts. Thus, aberrantly expressed miRNAs disrupt tightly controlled RNA networks in cancer cells. Our functional screening showed that expression of *miR-124-3p* was downregulated in pancreatic ductal adenocarcinoma (PDAC) tissues. Here, we aimed to investigate the anti-tumor roles of *miR-124-3p* in PDAC cells and to identify *miR-124-3p*-mediated oncogenic signaling in this disease. Ectopic expression of *miR-124-3p* inhibited cancer cell migration and invasion in PDAC cells. Moreover, restoration of *miR-124-3p* suppressed oncogenic signaling, as demonstrated by reduced phosphorylation of focal adhesion kinase, AKT, and extracellular signal-regulated kinase, in PDAC cells. Our *in silico* database analyses and luciferase reporter assays showed that two cell-surface matrix receptors, integrin α3 (*ITGA3*) and integrin β1 (*ITGB1*), were directly regulated by *miR-124-3p* in PDAC cells. Overexpression of *ITGA3* and *ITGB1* was confirmed in PDAC clinical specimens. Interestingly, a large number of cohort analyses from TCGA database showed that high expressions of *ITGA3* and *ITGB1* were significantly associated with poor prognosis of patients with PDAC. Knockdown of *ITGA3* and *ITGB1* by siRNAs markedly suppressed the migration and invasion abilities of PDAC cells. Moreover, downstream oncogenic signaling was inhibited by ectopic expression of *miR-124-3p* or knockdown of the two integrins. The discovery of anti-tumor miRNAs and miRNA-mediated oncogenic signaling may provide novel therapeutic targets for the treatment of PDAC.

## INTRODUCTION

Among human malignant tumors, pancreatic ductal adenocarcinoma (PDAC) is the most difficult cancer to treat, and the 5-year survival rate of patients with PDAC is approximately 5% [1]. Owing to the lack of an effective diagnostic strategy, most patients with PDAC have local invasion or distant metastasis [2]. Only about 15% of patients are candidates for surgical resection, and the prognosis of patients with inoperable cancer is very poor [3]. Currently developed combination chemotherapies have a limited therapeutic impact on patients with advanced-stage disease [4]. Elucidation of the molecular mechanisms of lethal PDAC may lead to the development of novel therapeutic strategies.

MicroRNAs (miRNAs) are short noncoding RNAs that act as fine-tuners of the expression of protein-coding or noncoding genes [5]. In recent years, miRNAs have been shown to have both oncogenic and tumor-suppressive functions based on a variety of studies [6]. Currently developed bioinformatics approaches have indicated that a single miRNA can regulate many protein-coding RNAs; conversely, more than 60% of protein-coding genes in the human genome are controlled by miRNAs [7]. Therefore, aberrantly expressed miRNAs can disrupt entire networks of protein-coding or noncoding genes and affect the pathogenesis of human cancers.

Screening for dysregulated miRNAs in cancer cells is a critical first step to elucidating aberrantly expressed genes and proteins involved in cancer pathogenesis [8]. In PDAC, we have sequentially identified anti-tumor miRNAs using RNA sequencing-based miRNA expression signatures [9–11]. Our previous studies showed that clustered miRNAs, e.g., *miR-375*, *miR-216a-5p*, *miR-216a-3p*, *miR-216b-5p*, *miR-216b-3p*, and *miR-217*, are significantly downregulated in PDAC tissues and act as anti-tumor miRNAs [9–11]. Moreover, overexpression of forkhead box Q1 (*FOXQ1*) and actin-binding protein anilin (*ANLN*) promote PDAC cell aggressiveness and are involved in PDAC pathogenesis [9, 10]. These findings suggested that analysis of novel cancer pathways mediated by anti-tumor miRNAs will provide new insights into the potential mechanisms underlying the aggressive course of PDAC.

In this study, we focused on *miR-124-3p* because our functional screening showed that restoration of *miR-124-3p* markedly inhibited oncogenic signaling in PDAC cells. Here, we aimed to investigate the anti-tumor roles of *miR-124-3p* and to identify *miR-124-3p*-regulated novel oncogenic pathways in PDAC cells. The *miR-124* family includes three members, *miR-124-1*, *miR-124-2* and *miR-124-3* on different human chromosomal loci, 8q23.1, 8p12.1 and 20p13.33, respectively. The mature sequences of the three *miR-124* family are exactly the same. Since their sequences are identical, we define the *miR-124* family as *miR-124-3p* in this study. The gene structure of the *miR-124* family and the chromosomal loci are shown in the Supplementary Figure 1.

Our present data showed that two cell-surface matrix receptors, integrin α3 (*ITGA3*) and integrin β1 (*ITGB1*), were directly regulated by *miR-124-3p* in PDAC cells. Recent studies demonstrated that dysregulation of the extracellular matrix (ECM) and integrin-mediated oncogenic signaling enhances cancer cell aggressiveness [12, 13]. Thus, *miR-124-3p*-based approaches for PDAC can be used to identify potential targets for the development of new therapeutic strategies.

## RESULTS

### Expression levels of *miR-124-3p* in PDAC clinical specimens and cell lines

Expression levels of *miR-124-3p* were validated using PDAC specimens (cancer tissues: n = 30 and normal pancreatic tissues: n = 12) and three PDAC cell lines (PANC-1, SW1990, and MIApaca-2). Backgrounds and clinicopathological characteristics of clinical samples are shown in Table 1A. Normal pancreatic tissues are shown in Table 1B.

**Table 1A:** Characteristics of patients with PDAC

Pancreatic ductal adenocarcinoma (PDAC)			(%)
Total number		30	
Average age (range), years		65.8 (42-79)	
Sex	Male	15	(50.0)
	Female	15	(50.0)
			
T category	pTis	1	(3.3)
	pT1	1	(3.3)
	pT2	1	(3.3)
	pT3	27	(90.1)
	pT4	0	(0)
			
N category	0	12	(40.0)
	1	18	(60.0)
			
M category	0	27	(90.0)
	1	3	(10.0)
			
Neoadjuvant chemotherapy	(-)	13	(43.3)
	(+)	17	(56.7)
			
Recurrence	(-)	8	(26.7)
	(+)	22	(73.3)

**Table 1B:** Characteristics of patients without PDAC

Normal pancreatic tissue			
Total number		12	
Average age (range), years		65.4 (42-85)	
Sex	Male	5	(41.7)
	Female	7	(58.3)

**Figure 1:**
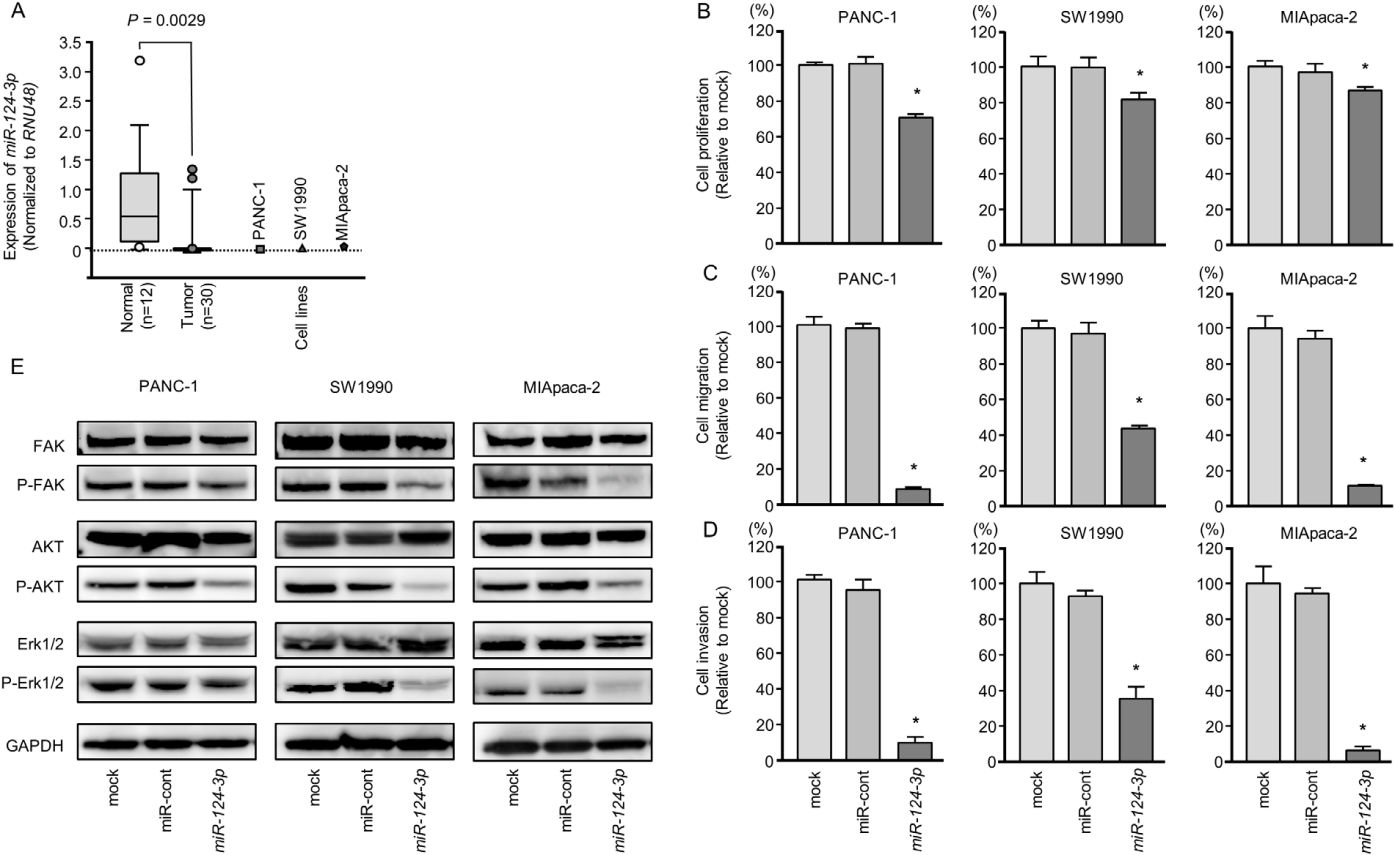
Anti-tumor functions of *miR-124-3p* in PDAC cell lines and decreased phosphorylation of the components of oncogenic signaling pathways **(A)** Expression levels of *miR-124-3p* in PDAC clinical specimens and cell lines were determined by qRT-PCR. Data were normalized to *RNU48* expression. ^*^, *P* < 0.0001. **(B)** Cell proliferation was determined by XTT assays 72 h after transfection with 10 nM *miR-124-3p*. ^*^, *P* < 0.0001. **(C)** Cell migration activity was determined by migration assays. ^*^, *P* < 0.0001. **(D)** Cell invasion activity was determined using Matrigel invasion assays. ^*^, *P* < 0.0001. **(E)** Gain of function *miR-124-3p* in PDAC cells reduced the phosphorylation of FAK, AKT, and Erk1/2. GAPDH was used as a loading control.

The expression levels of *miR-124-3p* were significantly lower in PDAC tissues than in normal pancreatic tissues (normalized to *RNU48*; *P* = 0.0029, Figure 1A). However, for clinicopathological factors (i.e., age, sex, neoadjuvant chemotherapy, and recurrence), there were no significant differences in the expression of *miR-124-3p*.

Expression levels of three cancer cell lines were markedly low compared to normal pancreatic tissues (Figure 1A).

### Effects of ectopic expression of *miR-124-3p* in PDAC cells

To investigate the anti-tumor roles of *miR-124-3p*, we performed gain-of-function studies using transfection of PANC-1, SW1990, and MIApaca-2 cells.

*In vitro* assays demonstrated that cell proliferation, migration, and invasion were significantly inhibited in *miR-124-3p* mimic transfectants compared those in with mock or miR-control transfectants (each, *P* < 0.0001; Figure 1B–1D), with particularly remarkable effects observed in migration and invasion assays. These results indicated that *miR-124-3p* had anti-tumor roles in PDAC cells and could be categorized as an anti-tumor miRNA. We performed flow cytometric analyses to determine the number of apoptotic cells following restoration of *miR-124-3p* expression. The apoptotic cell numbers (apoptotic and early apoptotic cells) were increased in *miR-124-3p* expression than in mock or miR-control transfectant cells (Supplementary Figure 2A and 2B). We showed that cleaved PARP expression was detected in restoration of *miR-124-3p* expression (Supplementary Figure 2C).

### Blocking of oncogenic signaling by ectopic expression of *miR-124-3p* in PDAC cells

Next, we analyzed whether oncogenic signaling pathways were affected using gain-of-function *miR-124-3p* in PDAC cell lines. FAK, AKT, and ERK1/2 were selected as intracellular carcinogenic signaling molecules, and the phosphorylated state of each protein was evaluated by western blotting. The levels of phospho-FAK, phospho-AKT, and phospho-Erk1/2 were blocked by *miR-124-3p* expression in PDAC cells (Figure 1E).

### Identification of *miR-124-3p*-regulated oncogenic pathways and targets in PDAC cells

To identify molecular oncogenic pathways and targets regulated by *miR-124-3p* in PDAC cells, we applied a combination of *in silico* database analyses and gene expression analyses in PDAC clinical specimens. Our strategy is shown in Supplementary Figure 3.

Using the TargetScan database 7.1, we annotated 4,450 putative target genes having *miR-124-3p* binding sites in their 3′- untranslated regions (UTRs). Gene expression data (GEO accession number: GSE15471) revealed that 2,148 genes were upregulated in PDAC clinical specimens (fold-change log_2_ > 1.0). Finally, we selected 435 genes that were putative oncogenic targets regulated by *miR-124-3p* in PDAC cells.

**Figure 2:**
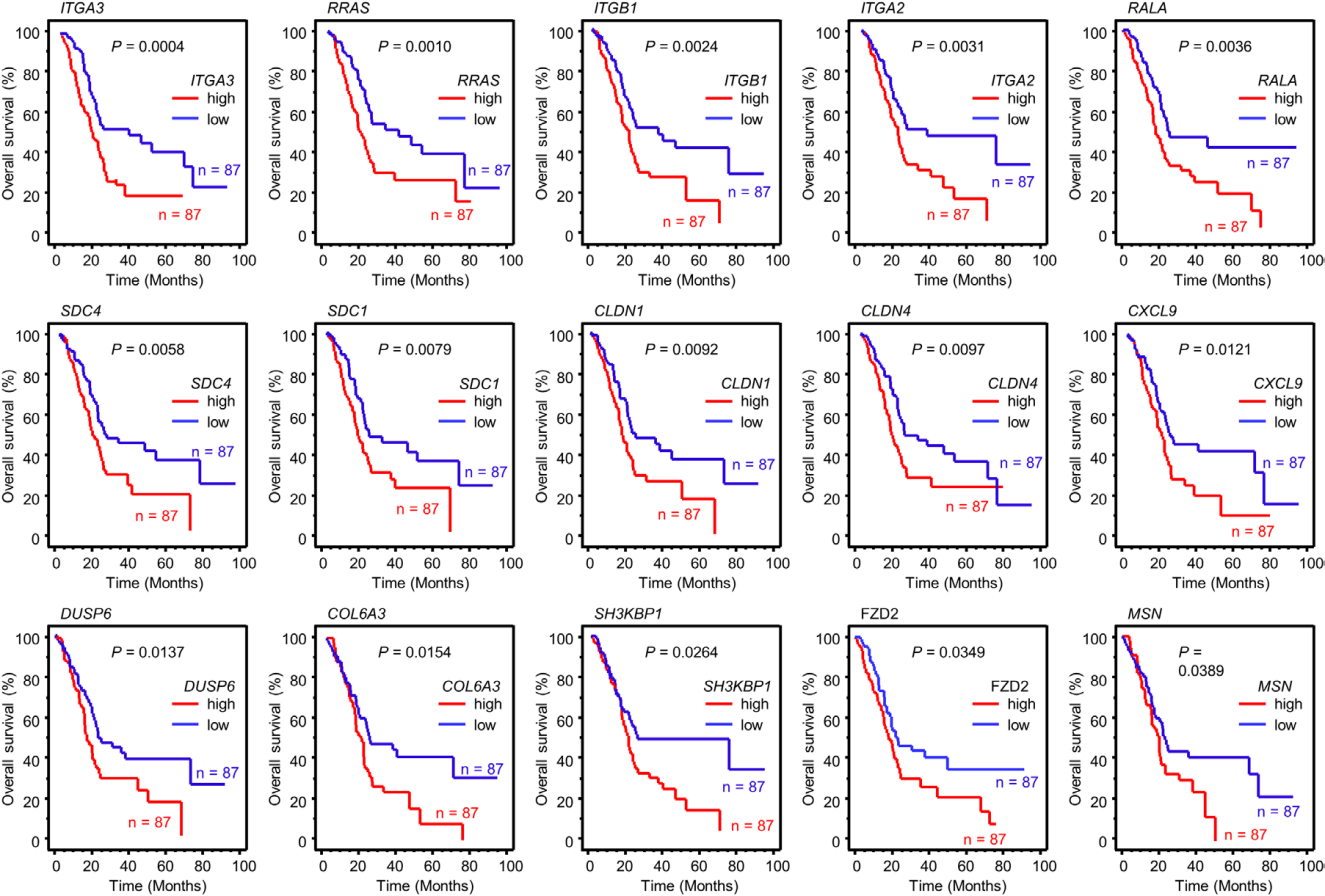
Kaplan-Meier analysis of *miR-124-3p*-regulated genes related to poor prognosis in PDAC Kaplan–Meier plots of overall survival with log-rank tests between those with high and low *miR-124-3p* expression and expression of 15 genes in the PDAC TCGA database.

We categorized 435 genes into existing molecular pathways using KEGG pathway analyses. The top 10 pathways contained 60 genes (Table 2A). We investigated the expression statuses of those 60 genes and the clinical significance of PDAC using the OncoLnc database (http://www.oncolnc.org/). Kaplan-Meier survival curves showed that high expression of 15 genes was associated with poor prognosis in PDAC (Table 2B, Figure 2).

**Table 2A:** Enriched KEGG pathways regulated by *miR-124-3p* on in silico analysis

KEGG ID	Pathways	*P* value	No. of genes	Genes
Kegg:04512	ECM-receptor interaction	4.1783E-11	13	*SDC1, ITGA1, SDC4, ITGA3, ITGA2, HSPG2, LAMC1, COL4A1, THBS2, LAMC2, ITGB1, COL5A1, COL6A3*
Kegg:05200	Pathways in cancer	1.0784E-09	21	*E2F3, TGFBR1, MMP2, GLI3, ITGA3, ITGA2, ETS1, AKT3, IL8, LAMC1, COL4A1, FZD2, TGFB1, MITF, PDGFRB, LAMC2, ITGB1, RALA, PLD1, CBL, CBLB*
Kegg:05145	Toxoplasmosis	3.5384E-06	10	*GNAI1, TLR4, AKT3, HSPA6, LAMC1, HLA-DPB1, TGFB1, IL10RA, LAMC2, ITGB1*
Kegg:04060	Cytokine-cytokine receptor interaction	5.5517E-06	14	*IL7R, CXCL9, TGFBR1, IL8, CCL2, TGFB1, IL10RA, PDGFRB, IFNAR2, CSF2RB, TNFRSF11B, TNFSF4, LIF, TNFSF11*
Kegg:04510	Focal adhesion	7.8047E-06	12	*ITGA1, ITGA3, ITGA2, AKT3, LAMC1, COL4A1, THBS2, PDGFRB, LAMC2, ITGB1, COL5A1, COL6A3*
Kegg:04630	Jak-STAT signaling pathway	2.4297E-05	10	*IL7R, AKT3, JAK3, SPRED1, IL10RA, IFNAR2, CSF2RB, CBL, LIF, CBLB*
Kegg:04514	Cell adhesion molecules (CAMs)	2.8873E-05	9	*CLDN1, SDC1, SDC4, MPZL1, HLA-DPB1, CLDN11, ITGB1, VCAN, CLDN4*
Kegg:04810	Regulation of actin cytoskeleton	7.0689E-05	11	*ITGA1, ARPC5, ARPC1B, ITGA3, MYH10, ITGA2, MSN, GNA13, RRAS, PDGFRB, ITGB1*
Kegg:04144	Endocytosis	0.00076292	9	*SH3KBP1, TGFBR1, HSPA6, TGFB1, RAB31, DAB2, PLD1, CBL, CBLB*
Kegg:04010	MAPK signaling pathway	0.00596607	9	*CACNA2D1, TGFBR1, AKT3, DUSP6, HSPA6, RRAS, TGFB1, PDGFRB, MAP3K8*

**Table 2B:** Candidate target genes regulated by *miR-124-3p*

Entrez gene	Gene symbol	Gene name	Target sites		GEO data FC(log2)	^*^TCGA_OncoLnc P-value
			Conserved sites	Poorly sites		
3675	*ITGA3*	integrin, alpha 3 (antigen CD49C, alpha 3 subunit of VLA-3 receptor)	1	0	1.329	0.0004
6237	*RRAS*	related RAS viral (r-ras) oncogene homolog	1	0	1.147	0.0010
3688	*ITGB1*	integrin, beta 1 (fibronectin receptor, beta polypeptide, antigen CD29 includes MDF2, MSK12)	2	0	1.687	0.0024
3673	*ITGA2*	integrin, alpha 2 (CD49B, alpha 2 subunit of VLA-2 receptor)	0	1	2.619	0.0031
5898	*RALA*	v-ral simian leukemia viral oncogene homolog A (ras related)	1	0	1.028	0.0036
6385	*SDC4*	syndecan 4	1	0	1.030	0.0058
6382	*SDC1*	syndecan 1	0	1	1.384	0.0079
9076	*CLDN1*	claudin 1	0	5	1.892	0.0092
1364	*CLDN4*	claudin 4	0	1	1.055	0.0097
4283	*CXCL9*	chemokine (C-X-C motif) ligand 9	0	1	1.294	0.0121
1848	*DUSP6*	dual specificity phosphatase 6	1	0	1.141	0.0137
1293	*COL6A3*	collagen, type VI, alpha 3	0	1	2.749	0.0154
30011	*SH3KBP1*	SH3-domain kinase binding protein 1	1	0	1.652	0.0264
2535	*FZD2*	frizzled family receptor 2	0	1	1.295	0.0349
4478	*MSN*	moesin	0	2	1.740	0.0389
7058	*THBS2*	thrombospondin 2	1	1	3.986	0.0572
7046	*TGFBR1*	transforming growth factor, beta receptor 1	1	0	1.567	0.0587
3918	*LAMC2*	laminin, gamma 2	0	1	2.761	0.0595
2113	*ETS1*	v-ets avian erythroblastosis virus E26 oncogene homolog 1	1	1	1.156	0.0674
161742	*SPRED1*	sprouty-related, EVH1 domain containing 1	1	0	1.539	0.1010
3587	*IL10RA*	interleukin 10 receptor, alpha	0	2	1.268	0.1380
3718	*JAK3*	Janus kinase 3	0	1	1.005	0.1670
11031	*RAB31*	RAB31, member RAS oncogene family	0	1	2.855	0.1680
4286	*MITF*	microphthalmia-associated transcription factor	2	0	1.117	0.2120
3976	*LIF*	leukemia inhibitory factor	1	2	1.161	0.2140
4628	*MYH10*	myosin, heavy chain 10, non-muscle	1	0	1.142	0.2170
868	*CBLB*	Cbl proto-oncogene B, E3 ubiquitin protein ligase	0	1	1.434	0.2380
8600	*TNFSF11*	tumor necrosis factor (ligand) superfamily, member 11	1	0	1.412	0.2850
6347	*CCL2*	chemokine (C-C motif) ligand 2	0	1	1.086	0.2990
10000	*AKT3*	v-akt murine thymoma viral oncogene homolog 3	2	0	1.023	0.3170
3339	*HSPG2*	heparan sulfate proteoglycan 2	0	1	1.080	0.3290
1289	*COL5A1*	collagen, type V, alpha 1	0	1	3.496	0.3300
10672	*GNA13*	guanine nucleotide binding protein (G protein), alpha 13	2	0	1.109	0.3300
781	*CACNA2D1*	calcium channel, voltage-dependent, alpha 2/delta subunit 1	1	0	1.619	0.3690
1439	*CSF2RB*	colony stimulating factor 2 receptor, beta, low-affinity (granulocyte-macrophage)	0	1	1.386	0.3940
1462	*VCAN*	versican	1	1	4.155	0.4380
4313	*MMP2*	matrix metallopeptidase 2 (gelatinase A, 72kDa gelatinase, 72kDa type IV collagenase)	0	2	2.531	0.4450
3575	*IL7R*	interleukin 7 receptor	0	1	1.444	0.4530
3115	*HLA-DPB1*	major histocompatibility complex, class II, DP beta 1	0	1	1.232	0.4540
7099	*TLR4*	toll-like receptor 4	0	3	1.095	0.5340
867	*CBL*	Cbl proto-oncogene, E3 ubiquitin protein ligase	3	2	1.058	0.5750
1282	*COL4A1*	collagen, type IV, alpha 1	1	1	2.357	0.5830
4982	*TNFRSF11B*	tumor necrosis factor receptor superfamily, member 11b	0	1	1.697	0.6000
3455	*IFNAR2*	interferon (alpha, beta and omega) receptor 2	0	1	1.072	0.6180
3672	*ITGA1*	integrin, alpha 1	0	2	1.561	0.6330
2770	*GNAI1*	guanine nucleotide binding protein (G protein), alpha inhibiting activity polypeptide 1	1	0	1.210	0.6980
10095	*ARPC1B*	actin related protein 2/3 complex, subunit 1B, 41kDa	1	0	1.572	0.7020
5010	*CLDN11*	claudin 11	0	1	2.540	0.7350
3915	*LAMC1*	laminin, gamma 1 (formerly LAMB2)	3	1	1.347	0.7430
5337	*PLD1*	phospholipase D1, phosphatidylcholine-specific	1	0	1.395	0.7630
5159	*PDGFRB*	platelet-derived growth factor receptor, beta polypeptide	0	1	1.799	0.7750
7292	*TNFSF4*	tumor necrosis factor (ligand) superfamily, member 4	0	1	1.575	0.7930
1601	*DAB2*	Dab, mitogen-responsive phosphoprotein, homolog 2 (Drosophila)	1	0	1.036	0.8810
7040	*TGFB1*	transforming growth factor, beta 1	0	1	1.423	0.8930
9019	*MPZL1*	myelin protein zero-like 1	1	0	1.002	0.8940
3576	*IL8*	interleukin 8	0	1	3.240	0.9220
1326	*MAP3K8*	mitogen-activated protein kinase kinase kinase 8	0	1	1.352	0.9280
10092	*ARPC5*	actin related protein 2/3 complex, subunit 5, 16kDa	0	1	1.038	0.9440
3310	*HSPA6*	heat shock 70kDa protein 6 (HSP70B')	0	1	1.347	0.9560
2737	*GLI3*	GLI family zinc finger 3	1	0	1.343	0.9890

^*^Kaplan-Meier analysis Log-rank.

P-value < 0.05 Poor prognosis with a high expression.

**Figure 3:**
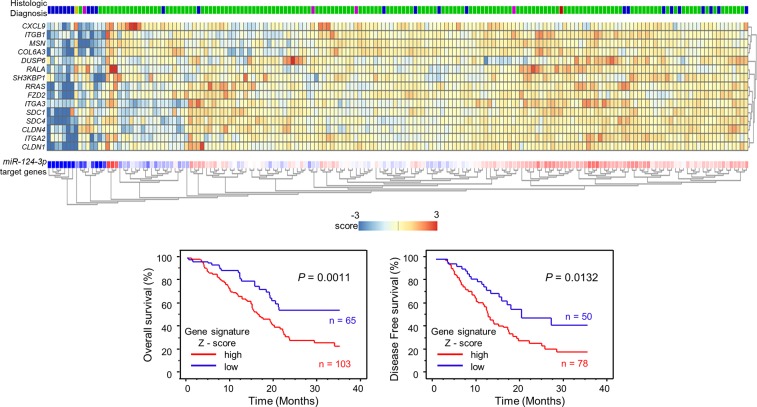
Combination analysis with heatmap of 15 target genes related to poor prognosis in PDAC Heatmap was created using analysis webcite “R2: Genomics Analysis and Visualization Platform (http://r2.amc.nl)”. Z - score was evaluated by a combination of *miR-124-3p* final target genes based on TCGA datasets. High group (mRNA Z-score > 0) and low group (mRNA Z-score ≤ 0) are displayed as Kaplan–Meier plots with log-rank tests.

Furthermore, we provided a heatmap gene visualization and validated as a prognostic ability of these 15 genes (Figure 3). As shown in Figure 3, patients with high gene signature expressions (Z-score > 0) were significantly poor Overall Survival (OS) and Disease Free Survival (DFS) rate than those with low gene signature expressions (Z-score ≤ 0) (OS; *P* = 0.0011, DFS; *P* = 0.0132, Figure 3). Combination analysis of *ITGA3* and *ITGB1* revealed that high expression of *ITGA3/ITGB1* (Z-score > 0) was predicted significantly poor OS and DFS rate than those with low expression of *ITGA3/ITGB1* (Z-score ≤ 0) (OS; *P* = 0.0037, DFS; *P* = 0.0162, Supplementary Figure 4). In this study, we focused on *ITGA3* and *ITGB1*.

### Overexpression of *ITGA3* and *ITGB1* in PDAC tissues and cell lines

**Figure 4:**
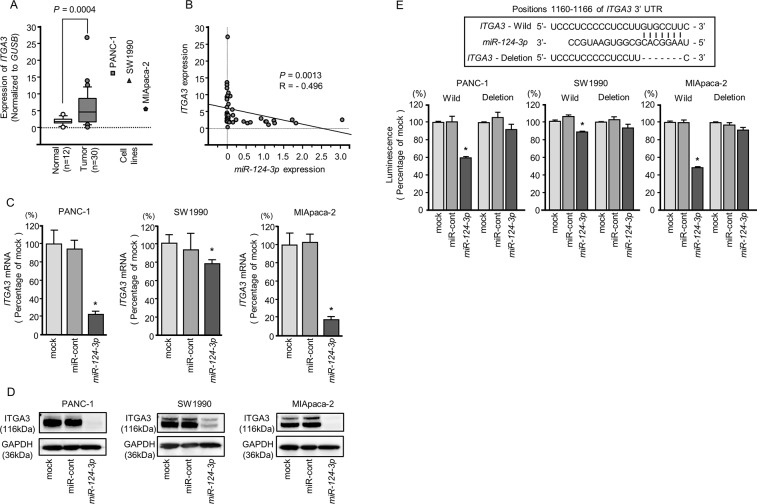
Direct regulation of *ITGA3* by *miR-124-3p* in PDAC cell lines **(A)** Expression levels of *ITGA3* in PDAC clinical specimens and cell lines were determined by qRT-PCR. Data were normalized to *GUSB* expression. **(B)** Expression levels of *ITGA3* and *miR-124-3p* were negatively correlated. **(C)** *ITGA3* mRNA expression in PDAC cell lines was evaluated by qRT-PCR 72 h after transfection with *miR-124-3p*. *GUSB* was used as an internal control. ^*^, *P* < 0.0001. **(D)** ITGA3 protein expression in PDAC cell lines was evaluated by western blot analysis 96 h after transfection with *miR-124-3p*. GAPDH was used as a loading control. **(E)** *miR-124-3p* binding sites in the 3′-UTR of *ITGA3* mRNA. Dual luciferase reporter assays using vectors encoding the putative *miR-124-3p* (positions 1160-1166) target site of the *ITGA3* 3′-UTR for both wild-type and deleted regions. Normalized data were calculated as ratios of *Renilla*/firefly luciferase activities. ^*^, *P* < 0.0001.

**Figure 5:**
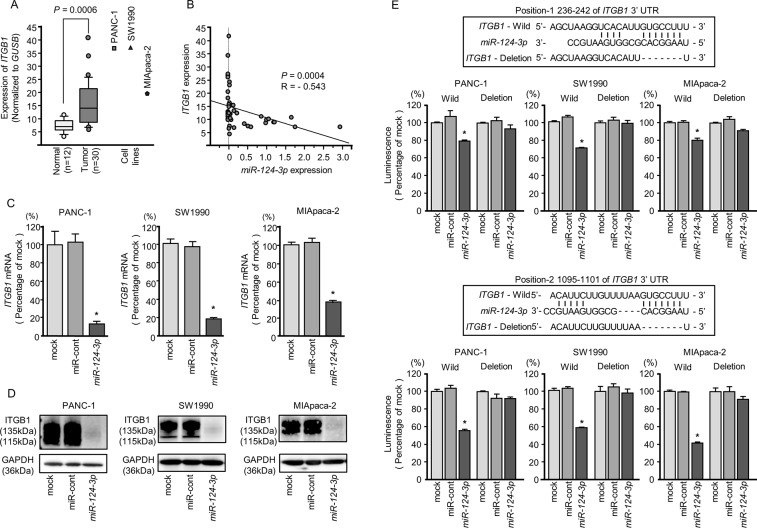
Direct regulation of *ITGB1* by *miR-124-3p* in PDAC cell lines **(A)** Expression levels of *ITGB1* in PDAC clinical specimens and cell lines were determined by qRT-PCR. Data were normalized to *GUSB* expression. **(B)** Expression levels of *ITGB1* and *miR-124-3p* were negatively correlated. **(C)** *ITGB1* mRNA expression in PDAC cell lines was evaluated by qRT-PCR 72 h after transfection with *miR-124-3p*. *GUSB* was used as an internal control. ^*^, *P* < 0.0001. **(D)** ITGB1 protein expression in PDAC cell lines was evaluated by western blot analysis 96 h after transfection with *miR-124-3p*. GAPDH was used as a loading control. **(E)** *miR-124-3p* binding sites in the 3′-UTR of *ITGB1* mRNA. Dual luciferase reporter assays using vectors encoding the putative *miR-124-3p* (positions 236–242 and 1095-1101) target sites of the *ITGB1* 3′-UTR for both wild-type and deleted regions. Normalized data were calculated as ratios of *Renilla*/firefly luciferase activities. ^*^, *P* < 0.0001.

We evaluated the expression levels of *ITGA3* and *ITGB1* in PDAC tissues (n = 30), normal pancreatic tissues (n = 12), and three PDAC cell lines (PANC-1, SW1990, and MIApaca-2). The expression levels of *ITGA3 and ITGB1* were significantly upregulated in PDAC tissues compared with normal pancreatic tissues (normalized to *GUSB*: *P* = 0.0004, Figure 4A and P = 0.0006, Figure 5A).

Negative correlations between *miR-124-3p* expression and *ITGA3* mRNA expression were analyzed by Spearman's rank test (*R* = −0.496, *P* < 0.0013, Figure 4B). Additionally, negative correlations between *miR-124-3p* expression and *ITGB1* mRNA expression were analyzed by Spearman's rank test (R = −0.543, *P* = 0.0004, Figure 5B).

### Direct regulation of *ITGA3* and *ITGB1* by *miR-124-3p* in PDAC cells

We performed qRT-PCR to validate *miR-124-3p* repression of *ITGA3* mRNA expression in PDAC cell lines. Our studies revealed that *ITGA3* mRNA was significantly reduced in *miR-124-3p* mimic transfectants in comparison with mock or miR-control transfectants (*P* < 0.0001, Figure 4C). Expression of ITGA3 protein was also repressed in the *miR-124-3p* mimic transfectants (Figure 4D). *In silico* analysis using TargetScan database 7.1 showed that the 3′-UTR of *ITGA3* harbored one binding site for *miR-124-3p* (Figure 4E). To determine whether *ITGA3* mRNAs had functional target site, we performed dual luciferase reporter assays. Compared with the miR-control, luminescence intensity was significantly reduced by transfection with *miR-124-3p* at *miR-124-3p* target sites, position 1160-1166 in the 3′-UTR of *ITGA3* (Figure 4E).

Similarly, we performed qRT-PCR to validate *miR-124-3p* repression of *ITGB1* mRNA expression in PDAC cell lines. Our studies revealed that *ITGB1* mRNA was significantly reduced in *miR-124-3p* mimic transfectants in comparison with mock or miR-control transfectants (*P* < 0.0001, Figure 5C). Expression of ITGB1 protein was also repressed in the *miR-124-3p* mimic transfectants (Figure 5D). *In silico* analysis using TargetScan database 7.1 showed that the 3′-UTR of *ITGB1* harbored two binding sites for *miR-124-3p* (Figure 5E). To determine whether *ITGB1* mRNAs had functional target sites, we performed dual luciferase reporter assays. Compared with the miR-control, luminescence intensity was significantly reduced by transfection with *miR-124-3p* at *miR-124-3p* target sites, both position-1 236-242 in the 3′-UTR of *ITGB1* (Figure 5E, upper) and position-2 1095-1101 in the 3′-UTR of *ITGB1* (Figure 5E, lower)

### Effects of *ITGA3* and *ITGB1* knockdown in PDAC cells

To validate the oncogenic functions of *ITGA3* and *ITGB1* in PDAC cells, we performed knockdown assays using siRNAs (si-*ITGA3* and si-*ITGB1*).

**Figure 6:**
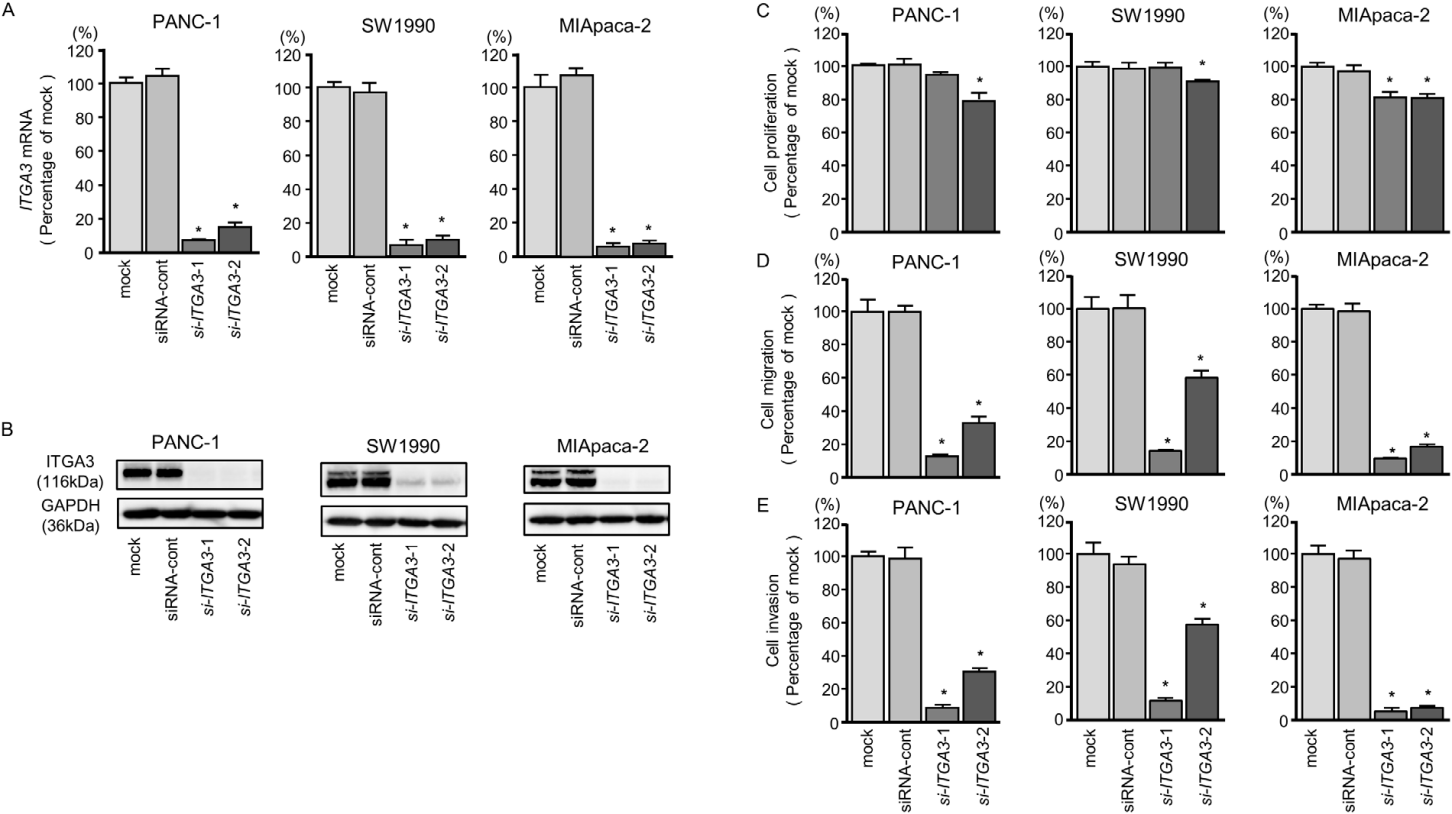
*ITGA3* mRNA and protein expression after si-*ITGA3* transfection and effects of *ITGA3* silencing in PDAC cell lines **(A)** *ITGA3* mRNA expression in PDAC cell lines was evaluated by qRT-PCR 72 h after transfection with si-*ITGA3*-1 or si-*ITGA3*-2. *GUSB* was used as an internal control. **(B)** ITGA3 protein expression in PDAC cell lines was evaluated by western blot analysis 96 h after transfection with si-*ITGA3-1* and si-*ITGA3-2*. GAPDH was used as a loading control. **(C)** Cell proliferation was determined using XTT assays 72 h after transfection with 10 nM si-*ITGA3*-1 or si-*ITGA3*-2. ^*^, *P* < 0.0001. **(D)** Cell migration activity was determined using migration assays. ^*^, *P* < 0.0001. **(E)** Cell invasion activity was determined by Matrigel invasion assays. ^*^, *P* < 0.0001.

**Figure 7:**
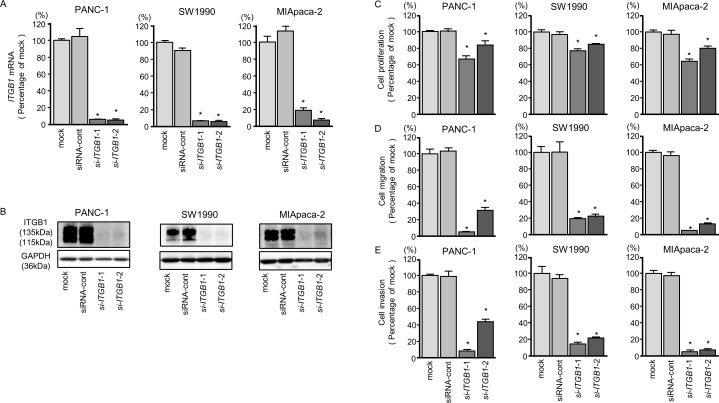
*ITGB1* mRNA and protein expression after si-*ITGB1* transfection and effects of *ITGB1* silencing in PDAC cell lines **(A)** *ITGB1* mRNA expression in PDAC cell lines was evaluated by qRT-PCR 72 h after transfection with si-*ITGB1*-1 or si-*ITGB1*-2. *GUSB* was used as an internal control. **(B)** ITGB1 protein expression in PDAC cell lines was evaluated by western blot analysis 96 h after transfection with si-*ITGB1-1* and si-*ITGB1-2*. GAPDH was used as a loading control. **(C)** Cell proliferation was determined with the XTT assays 72 h after transfection with 10 nM si-*ITGB1*-1 or si-*ITGB1*-2. ^*^, *P* < 0.0001. **(D)** Cell migration activity was determined using migration assays. ^*^, *P* < 0.0001. **(E)** Cell invasion activity was determined by Matrigel invasion assays. ^*^, *P* < 0.0001.

First, we evaluated the knockdown efficiency of si-*ITGA3* and si-*ITGB1* transfection in PDAC cell lines. In this study, we used two types of si-*ITGA3* (si-*ITGA3*-1 and si-*ITGA3*-2) and si-*ITGB1* (si-*ITGB1*-1 and si-*ITGB1*-2). Our data showed that all siRNAs effectively reduced *ITGA3* and *ITGB1* mRNA levels of expression in PDAC cell lines (Figures 6A and 7A). Furthermore, all siRNAs effectively reduced ITGA3 and ITGB1 protein levels of expression in PDAC cell lines (Figures 6B and 7B).

Moreover, cancer cell proliferation, migration, and invasion abilities were suppressed by knockdown of these genes (Figures 6C-6E and 7C-7E). The apoptotic cell numbers were increased in si-*ITGA3* or si-*ITGB1* transfectant cells than in mock or siRNA-control transfectant cells (Supplementary Figure 2A and 2B). We showed that cleaved PARP expression was detected in transfectant cells into si-*ITGA3* or si-*ITGB1* (Supplementary Figure 2C).

### Expression of ITGA3 and ITGB1 in PDAC clinical specimens and its clinical significance

**Figure 8:**
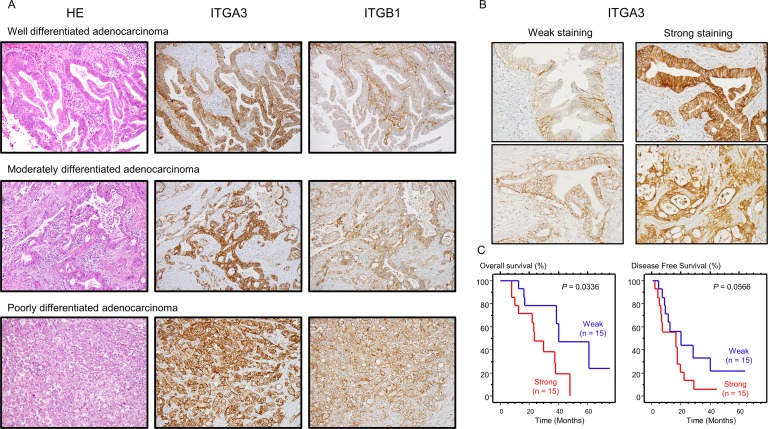
Expression levels of ITGA3/ITGB1 as determined by immunohistochemical staining in PDAC specimens Immunohistochemical staining of ITGA3/ITGB1 in PDAC specimens. **(A)** All differentiated types of PDAC (well, moderately, poorly) showed immunoreactivity (left panel: hematoxylin-eosin staining, middle panel: ITGA3 staining, right panel: ITGB1 staining, original magnification, 200×). **(B)** Immunostaining of ITGA3 was classified by Weak (left panel) and Strong (right panel). The expression of ITGA3 was evaluated using high-power microscopy (400×). **(C)** Kaplan-Meier curves for OS and DFS rates based on ITGA3 expression in 30 patients with PDAC. P-values were calculated using the log-rank test.

Next, we confirmed the expression of ITGA3 and ITGB1 proteins in PDAC clinical specimens using immunohistochemistry (IHC). In total, 30 PDAC specimens were evaluated. ITGA3 showed intracellular and membrane immunoreactivity in PDAC tissue, whereas ITGB1 showed medium membrane immunoreactivity in PDAC tissue (Figure 8A). For ITGA3-IHC staining, we categorized into “Weak” and “Strong” depending on the intensity of staining. (Figure 8B), OS and DFS were evaluated by Kaplan-Meier analysis, OS was shown that strong staining has poor prognosis with significant (*P* = 0.0336, Figure 8C). Details of clinicopathologic factors was shown in Supplementary Table 1. High ITGA3 expression was significantly associated with increased lymph node metastasis. In contrast, there were no significant relationships between ITGB1-IHC staining.

Finally, we examined the relationships between integrin expression and clinicopathological factors using TCGA database. There were no significant differences in terms of T, N, M, and TNM stages. However, high expression of *ITGA3* was significantly associated with recurrence. In addition, DFS was significantly shorter in the high expression group (Supplementary Figure 5). In contrast, there were no significant relationships between *ITGB1* expression and clinicopathological factors.

## DISCUSSION

PDAC is the most lethal type of gastrointestinal cancer because it often does not cause any signs or symptoms in the early stages [1]. Moreover, there are no effective treatment strategies for patients with advanced stage PDAC, and the prognosis is extremely poor. Elucidation of the aggressive nature of PDAC based on current genomic approaches will provide important insights into novel treatment strategies for this disease. We have sequentially identified novel oncogenic RNA networks based on PDAC miRNA analyses [10]. Our recent studies showed that the *miR-216* family and *miR-217* were downregulated in PDAC tissues and that these miRNAs acted as anti-tumor miRNAs by targeting *FOXQ1* and *ANLN*, respectively [9, 10]. Additionally, overexpression of *FOXQ1* and *ANLN* has been shown to promote cancer cell aggressiveness and is involved in PDAC pathogenesis [9, 10].

In this study, we focused on *miR-124-3p* because miRNA functional assays showed that ectopic expression of *miR-124-3p* dramatically inhibited cancer cell migration and invasion by blocking several oncogenic signals. Previous studies have indicated that *miR-124-3p* is a bona fide anti-tumor miRNA in several cancers, e.g., glioblastoma, esophageal cancer, hepatocellular carcinoma [14–16]. These studies showed that overexpression of *miR-124-3p* inhibited cancer cells migration and invasion abilities through targeting several oncogenes, e.g., *IQGAP1*, *LAMC1*, *ITGB1*, *STAT3*, *SP1* [14–16]. Other studies indicated that promoter region of *miR-124* genes (*miR-124-1*, *miR-124-2* and *miR-124-3*) were methylated in cancer cells [17, 18]. In PDAC cells, all member of *miR-124* family were highly methylated in PDAC tissues compared with in non-cancerous tissues by pyrosequencing analysis [19]. Epigenetic modification of *miR-124* genes is greatly involved in silencing *miR-124-3p* expression in several cancer cells. Our data confirmed that *miR-124-3p* acted as an anti-tumor miRNA in PDAC cells, consistent with previous reports [19].

Next, we aimed to discover molecular networks controlled by anti-tumor *miR-124a-3p* in PDAC cells. To identify *miR-124-3p*-regulated networks, we applied *in silico* database analyses depending on a strategy we created independently [9–11]. Several pathways were found to be regulated by *miR-124-3p* in PDAC cells, including ECM-receptor interactions, pathways in cancer, cytokine-cytokine receptor interactions, focal adhesion, and actin cytoskeleton regulation. Finally, a total of 60 genes were identified as putative targets of *miR-124-3p* in PDAC cells. We further analyzed the genes involved in these pathways and PDAC pathogenesis using TCGA database. Importantly, among 60 putative targets, high expression of 15 genes (*ITGA3*, *RRAS*, *ITGB1*, *ITGA2*, *RALA*, *SDC4*, *SDC1*, *CLDN1*, *CLDN4*, *CXCL9*, *DUSP6*, *COL6A3*, *SH3KBP1*, *FZD2*, and *MSN*) was significantly associated with poor prognosis in patients with PDAC. Interestingly, many of these genes were involved in ECM/integrin signaling. The ECM is an assembly of extracellular molecules secreted by cells and provides structural and biochemical support to surrounding cells [20, 21]. Many studies have indicated that aberrant expression of ECM-related genes and activation of integrin-mediated signaling enhance cancer cell migration and invasion abilities in several cancers [22, 23]. Moreover, dysregulation of these genes is involved in the pathogenesis of PDAC [24], and detailed analyses of the functional significance of these genes in PDAC cells are necessary.

Integrins are a family of transmembrane cell adhesion receptors that are responsible for mutual cell-to-cell or cell-to-ECM, e.g., laminin, collagen, elastin, and fibronectin, communication [25]. Integrin receptors composed of specific two subunits (α and β) and up to 24 distinct integrin receptors have been reported [26]. Overexpression of integrins and activation of integrin-mediated oncogenic signaling have been reported in several cancers, affecting neighboring cancer cells or surrounding stromal cells and enhancing cancer cell aggressiveness and metastasis [27, 28]. Our recent studies have shown that anti-tumor miRNAs, e.g., the *miR-29* family, *miR-150*s, the *miR-199* family, *miR-218*, and *miR-223*, directly regulate several integrins, and these ligands and ectopic expression of these miRNAs have dramatically prevented cancer cell malignancy. [12, 13, 29–31]

Our present data showed that both *ITGA3* and *ITGB1* were directly regulated by anti-tumor *miR-124-3p* in PDAC cells, and knockdown of these genes or ectopic expression of *miR-124-3p* significantly blocked cancer cell aggressiveness. Moreover, overexpression of *ITGA3* and *ITGB1* was involved in PDAC pathogenesis. *ITGA3* and *ITGB1* form a specific type of integrin receptor (*ITGA3*/*ITGB1*), and dysregulation of *ITGA3*/*ITGB1*-mediated signaling enhances cancer cell aggressiveness in several types of cancer [32–34]. *ITGB1* can form 18 receptor heterodimers with various partners (integrin α-subunits), and these receptors regulate numerous signaling pathways under both physiological and pathophysiological conditions [35]. Overexpression of *ITGB1* has been frequently observed in several cancers, including PDAC [36]. Therefore, blocking *ITGB1*, e.g., using small molecules or antibodies, may be an effective approach for the treatment of lethal PDAC [37]. Furthermore, our findings revealed that *ITGA3* affected PDAC prognosis by altering recurrence and DFS. Anti-tumor *miR-124-3p* regulates important intracellular pathways for pancreatic cancer via dual *ITGA3*/*ITGB1*. Identification of novel anti-tumor miRNA-mediated RNA networks may contribute to the development of new therapeutic strategies. *ITGA2* is also reported as a partner subunit of *ITGB1* [38]. Our analysis showed that *ITGA2* was identified as *miR-124-3p* target and its expression was associated with poor prognosis of the patients with PDAC (Supplementary Figure 6). Previous studies showed that blocking of *ITGA2* inhibited cancer cell aggressiveness [39]. Therefore, it is necessary to analyze the functional significance of *ITGA2* in PDAC pathogenesis.

In conclusion, the anti-tumor roles of *miR-124-3p* were achieved in PDAC cells through inhibition of several oncogenic signaling pathways. Oncogenic pathways and targets regulated by *miR-124-3p* were involved in PDAC molecular pathogenesis. This is the first report demonstrating that the pivotal oncogenic receptors *ITGA3* and *ITGB1* were directly controlled by *miR-124-3p* in PDAC cells. Overexpression of these receptors was significantly associated with poor prognosis in patients with PDAC. Novel approaches based on anti-tumor miRNA-mediated oncogenic pathways and targets may contribute to the development of effective therapies for PDAC.

## MATERIALS AND METHODS

### Clinical pancreatic specimens and PDAC cell lines

In total, 30 PDAC specimens were obtained from patients who had undergone curative surgical resection at Kagoshima University Hospital from 1997 to 2016. Twelve normal pancreatic tissue specimens were collected from noncancerous regions. Frozen specimens were used for gene expression analysis, and paraffin sections were used for immunohistochemistry. The samples were staged according to the American Joint Committee on Cancer–Union Internationale Contre le Cancer (UICC) TNM classification [40].

The present study was approved by the Institutional Review Board of Kagoshima University; prior written informed consent and approval was given by each patient.

Three human PDAC cell lines (PANC-1, SW1990, and MIApaca-2) obtained from RIKEN Cell Bank (Tsukuba, Ibaraki, Japan) and the American Type Culture Collection (Manassas, VA, USA) were used for this study.

RNA containing miRNAs was extracted using ISOGEN (NIPPON GENE, Toyama, Japan), according to the manufacturer's instructions.

### Quantitative real-time polymerase chain reaction (qRT-PCR)

TaqMan qRT-PCR probes and primers were obtained from Thermo Fisher Scientific (Waltham, MA, USA) as follows: *miR-124-3p* (product ID: 000446; Thermo), *ITGA3* (product ID: Hs01076879_m1), *ITGB1* (product ID: Hs01127536_m1) and *ITGA2* (product ID: Hs00158127_m1). *GUSB* (product ID: Hs99999908_m1) and *RNU48* (product ID: 001006) were used as internal controls. The procedure for qRT-PCR was described in previous studies [30, 41].

### Transient transfection of mature miRNAs and small interfering RNAs (siRNAs) into cancer cells

Pre-miR miRNA precursors and siRNAs were purchased from Thermo Fisher Scientific, as follows: *miR-124-3p* (product ID: PM 10245), negative control miRNA (product ID: AM 17111), two *ITGA3* siRNAs (product IDs: HSS105529 and HSS179967), two *ITGB1* siRNAs (product IDs: HSS105559 and HSS105561), and negative control siRNA (product ID: D-001810-10). Lipofectamine RNAiMAX and Lipofectamine 2000 (Thermo Fisher Scientific) were used for transfection into PDAC cell lines. The transfection efficiencies of miRNA in cancer cells were calculated as described in previous studies [42].

### Cell proliferation, migration, and invasion assays

Cell proliferation was evaluated using a Cell Proliferation Kit II (Roche Applied Sciences, Penzberg, Germany). Cell migration was evaluated using BD Falcon Cell Culture Inserts (BD Biosciences, Franklin Lakes, NJ, USA), and cell invasion was evaluated using Corning BioCoat Matrigel Invasion Chambers (Corning, NY, USA). The protocols for functional assays were described previously [11, 13, 30].

### Identification of *miR-124-3p* targets in PDAC cells

To identify *miR-124-3p* target genes, we performed a combination of genome-wide gene expression analyses and *in silico* database analyses as described in previous studies [9–11, 43].

We selected putative miRNA target genes using the Target Scan Human 7.1 database (http://www.targetscan.org/vert_71), GEO microarray database (https://www.ncbi.nlm.nih.gov/geo//; accession number GSE15471), to analyze gene expression levels in 36 PDAC samples versus 36 normal pancreatic tissue samples. Finally, The Cancer Genome Atlas (TCGA) database (https://cancergenome.nih.gov/) was used for analysis of gene expression in PDAC. The strategy for selecting target genes in this study is shown in Supplementary Figure 3.

### TCGA database analysis of PDAC samples

To investigate the clinical significance of the expression status of putative targets of *miR-124-3p* in patients with PDAC, we analyzed TCGA datasets (https://gdc.nci.nih.gov/). A large amount of cohort data was retrieved from cBioPortal (http://www.cbioportal.org/) and OncoLnc (data downloaded on November 1, 2017). Detailed information on the method is described in a previous paper [44–45]. The Z-scores of target genes mRNA expression data and clinical sample information corresponding to PDAC patients were collected from cBioPortal. R2: Genomics Analysis and Visualization Platform (http://r2.amc.nl) was used to create a heatmap. Furthermore, Z- score was evaluated by a combination of each genes sets. High group (mRNA Z-score > 0) and low group (mRNA Z-score ≤ 0) were analyzed by Kaplan–Meier survival curves and log-rank statistics.

### Plasmid construction and dual-luciferase reporter assay

Wild-type or deletion-type sequences of the 3′-UTR of *ITGA3* and *ITGB1* in *miR-124-3p* target sites were inserted into the psiCHECK-2 vector (C8021; Promega, Madison, WI, USA). The procedure for dual luciferase reporter assays was described previously [11, 12, 42].

### Western blot analyses

Antibodies used in the phosphorylation pathway were purchased from Cell Signaling Technology (Danvers, MA, USA) as follows: anti-focal adhesion kinase (FAK) antibodies (product ID: #3285), anti-phospho-FAK antibodies (product ID: #8556), anti-AKT antibodies (product ID: #4691), anti-phospho-AKT antibodies (product ID: #4060), anti-extracellular signal-regulated kinase (Erk) 1/2 antibodies (product ID: #4695), and anti-phospho-Erk1/2 antibodies (product ID: #4370).

To investigate the expression of *miR-124-3p* targets, anti-ITGA3 antibodies (HPA008572; Sigma-Aldrich, St. Louis, MO, USA) and anti-ITGB1 antibodies (#9699; Cell Signaling Technology) were used for western blotting, PARP antibody (product ID: 9542; Cell Signaling Technology, Danvers, USA) was used for apoptosis assay, usage was according to each data sheet. Anti-glyceraldehyde 3-phosphate dehydrogenase (GAPDH) antibodies (product ID: SAF6698; Wako, Osaka, Japan) were used as an internal loading control for western blotting. A detailed description of the western blotting procedure was published elsewhere [11].

### Immunohistochemistry

Tissue sections were incubated overnight at 4°C with anti-ITGA3 antibodies diluted 1:200 (HPA008572; Sigma-Aldrich, St. Louis, MO, USA) and anti-ITGB1 antibodies diluted 1:100 (#9699; Cell Signaling Technology). The IHC score was calculated by judging the percentage of positively stained plasma membrane in cancer cells. The scores ranged from 1 to 3 (1; <5%, 2; 5–50%, and 3; >50% immunoreactive cells). Scores 1 and 2 were considered to indicate “weak” expression, while scores 3 indicated “Strong” expression of ITGA3.

### Statistical analysis

Mann-Whitney U-tests were used for analysis between two groups, and Bonferroni-adjusted Mann-Whitney U-tests were used for analysis among multiple groups. Correlations between the expression of *miR-124-3p* and *ITGA3/ITGB1* were evaluated using Spearman's rank tests. Overall survival (OS) and disease-free survival (DFS) after surgery were analyzed using Kaplan–Meier curves and evaluated with log-rank tests. We used Expert StatView software (version 5.0 SAS Institute Inc., Cary, NC, USA) for these analyses.

## SUPPLEMENTARY MATERIALS FIGURES AND TABLE


