# Supplementary material for: Involvement of anti-tumor miR-124-3p and its targets in the pathogenesis of pancreatic ductal adenocarcinoma: direct regulation of ITGA3 and ITGB1 by miR-124-3p
Source: Oncotarget. 2018 Jun 22;9(48):28849–65. doi: 10.18632/oncotarget.25599 (PMC6034741; doi:10.18632/oncotarget.25599)
Supplement: Supplementary file 1 [file oncotarget-09-28849-s001.pdf]

Involvement of anti-tumor *miR-124-3p* and its targets in the pathogenesis of pancreatic ductal adenocarcinoma: direct regulation of *ITGA3* and *ITGB1* by *miR-124-3p*

SUPPLEMENTARY MATERIALS

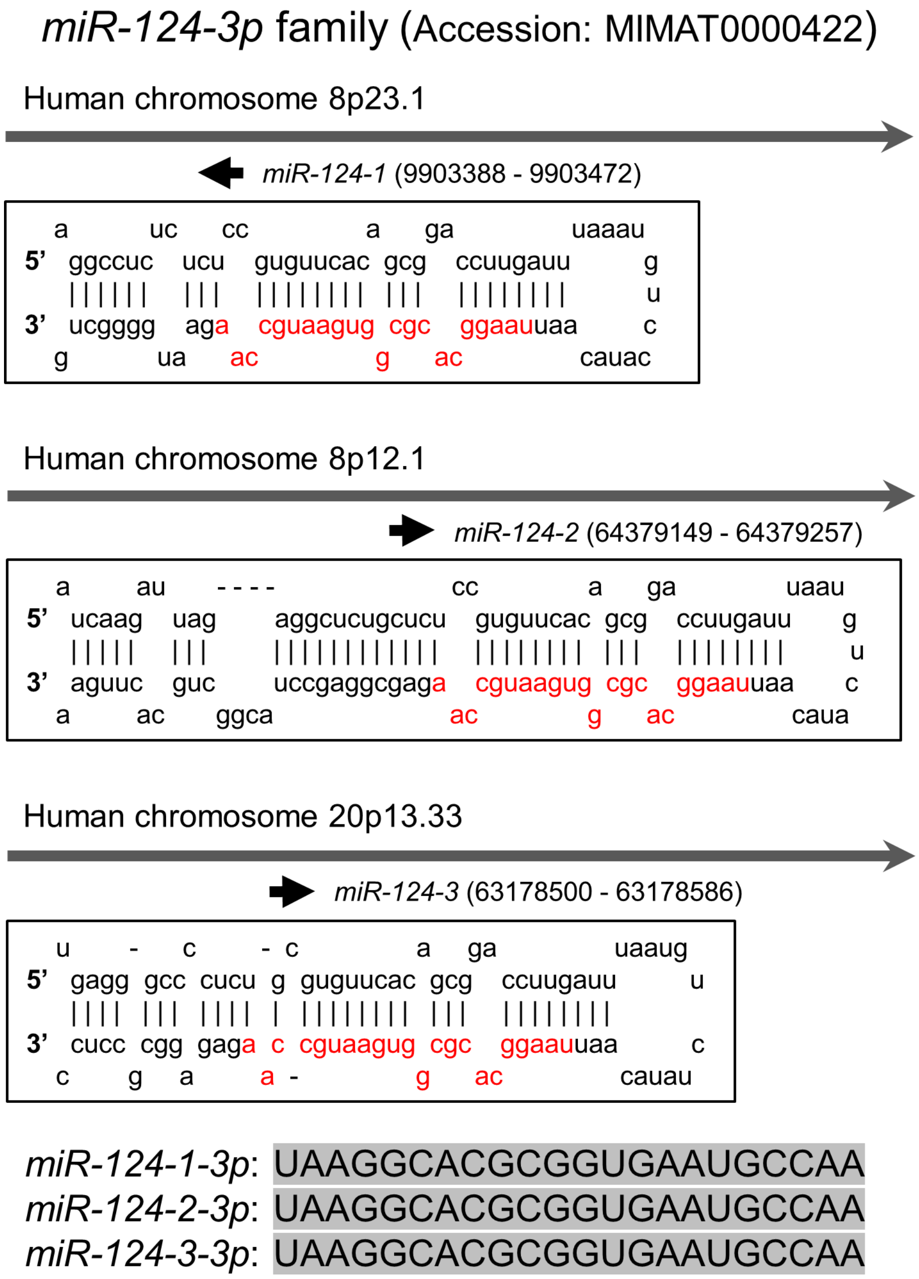

Supplementary Figure 1: Schematic representation of the human *miR-124* family in chromosomal location. Information on the sequence was obtained from miRbase (http://www.mirbase.org/).

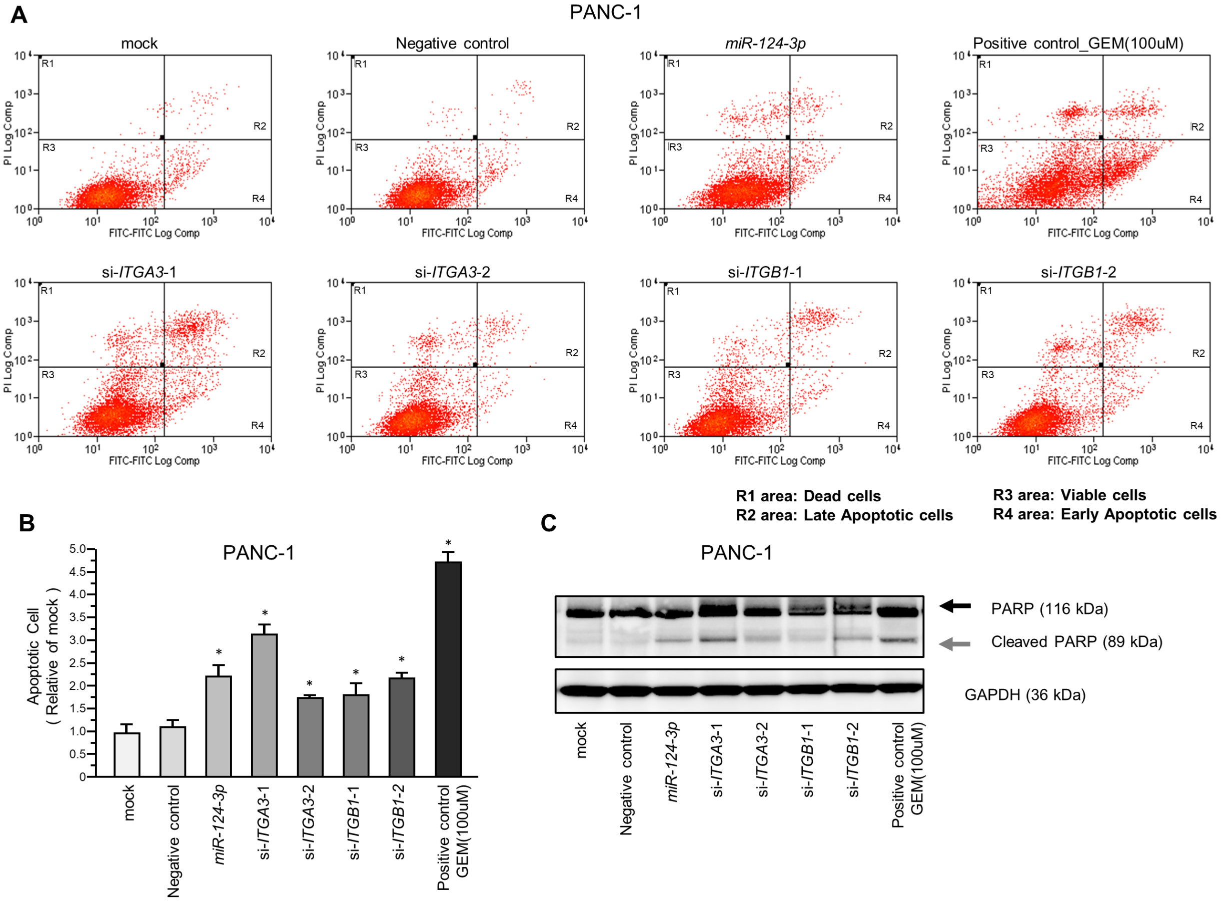

Supplementary Figure 2: Effects of *miR-124-3p*, si-*ITGA3* and si-*ITGB1* on apoptosis. **(A)** Apoptosis assays were performed using FITC Annexin V Apoptosis Detection Kit (BD Biosciences, Bedford, MA, USA) by flow cytometry (CyAn ADP analyzer; Beckman Coulter, Brea, CA, USA). Summit 4.3 software (Beckman Coulter) was used for analysis, early apoptotic cells are in area R4 and late apoptotic cells are in area R2. **(B)** The normalized ratios of apoptotic cells are shown in the histogram. Gemcitabine Hydrochloride (100uM) was used as positive control (GEM; Tokyo Chemical Industry, Tokyo, Japan), $^{*}P < 0.05$. **(C)** Western blot analyses for apoptotic markers (cleaved PARP) in PANC-1 cells. GAPDH was used as a loading control.

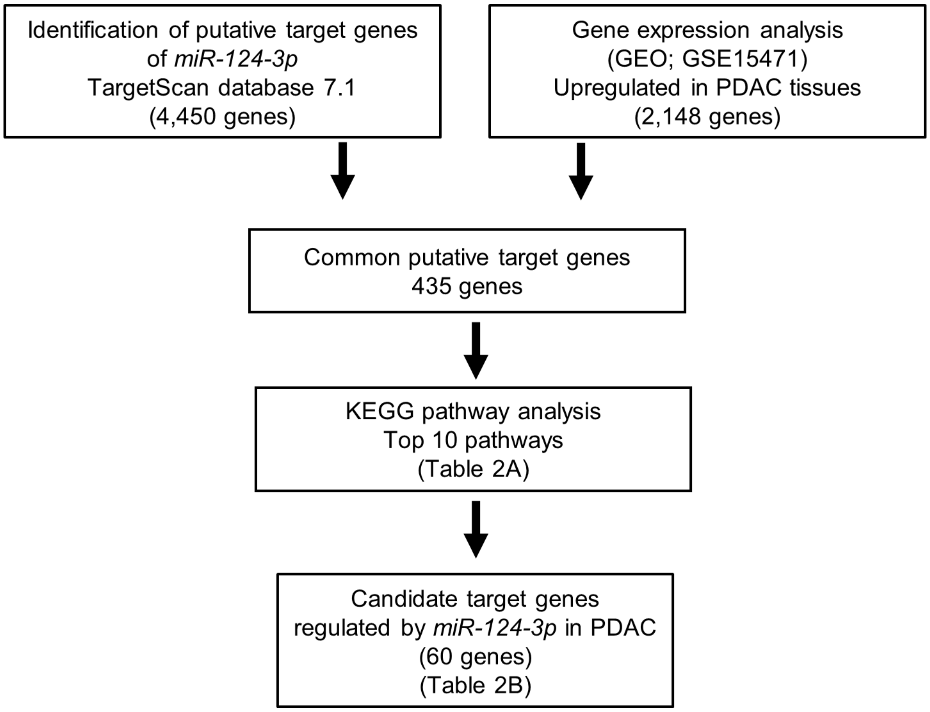

Supplementary Figure 3: The strategy for selection of *miR-124-3p* candidate target genes. The approach to identifying *miR-124-3p* target genes. We used *in silico* analysis of genome-wide expression, TargetScan, GEO database analysis, and KEGG enrichment analysis (http://genecodis.cnb.csic.es/).

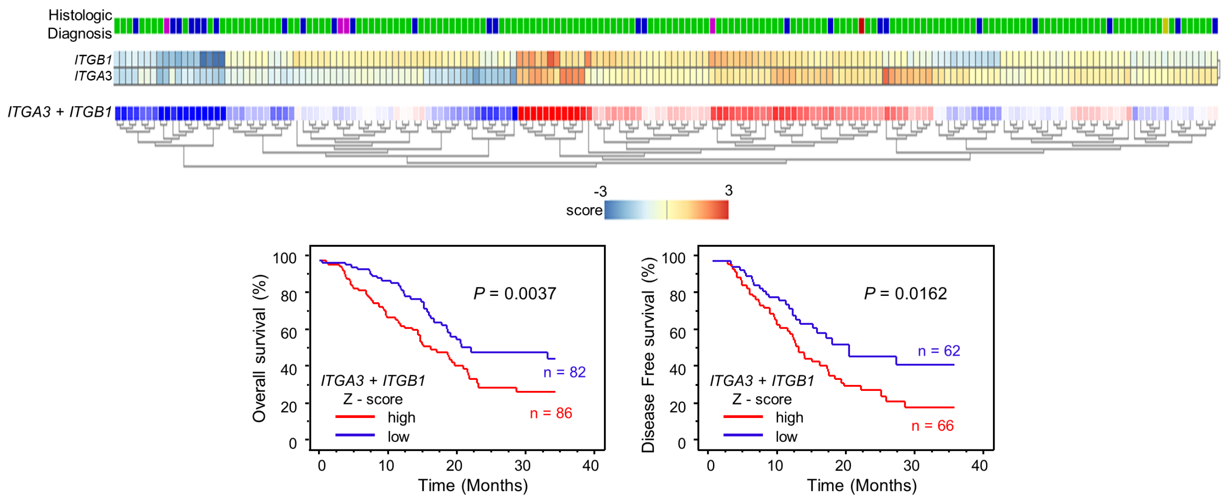

Supplementary Figure 4: Prognostic analysis of PDAC patients using *ITGA3* and *ITGB1* mRNA combination heatmap. Heatmap was created using analysis of "R2: Genomics Analysis and Visualization Platform (http://r2.amc.nl)". Z- score was evaluated by a combination of *ITGA3* and *ITGB1* mRNA based on TCGA datasets. High group (mRNA Z-score > 0) and low group (mRNA Z-score ≤ 0) are displayed as Kaplan–Meier plots with log-rank tests.

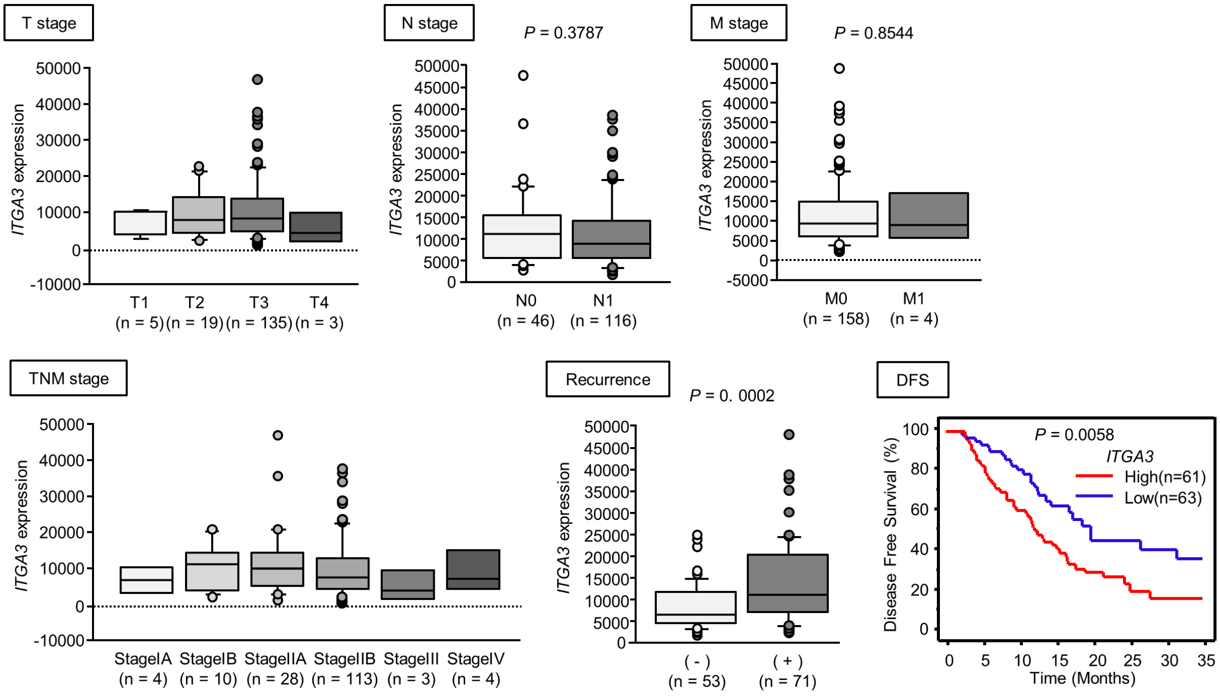

Supplementary Figure 5: Analysis of clinicopathological factors related to *ITGA3* in TCGA database. Clinicopathological factors in TCGA database were extracted from cBioportal, and each factor was analyzed according to *ITGA3* expression.

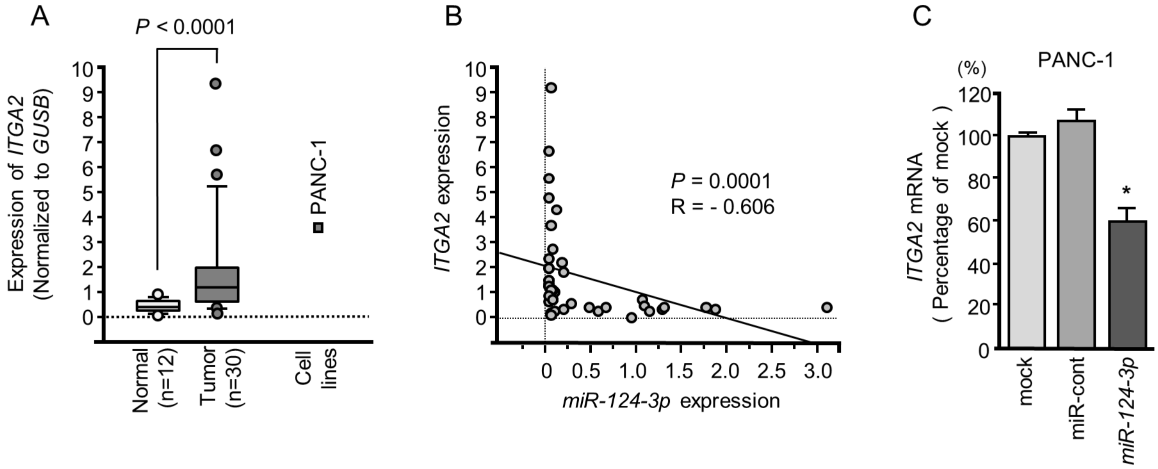

Supplementary Figure 6: Expression of *ITGA2* in PDAC clinical samples, downregulated by *miR-124-3p* in PANC-1 cells. **(A)** Expression levels of *ITGA2* in PDAC clinical specimens and PANC-1 cells were determined by qRT-PCR. Data were normalized to GUSB expression. **(B)** Expression levels of *ITGA2* and *miR-124-3p* were negatively correlated. **(C)** *ITGA2* mRNA expression in PDAC cell lines was evaluated by qRT-PCR 72 h after transfection with *miR-124-3p*. *GUSB* was used as an internal control. *, $P < 0.0001$.

Supplementary Table 1: Clinicopathological factors of IHC-ITGA3 in PDAC (n =30)

Characteristic	ITGA3		*P*
	Weak (n = 15)	Strong (n = 15)	
Age (y)[a]			[b]NS
≥ 60	12	11	
< 60	3	4	
Gender (n)			
Male (15)	6	9	NS
Female (15)	9	6	
T stage			
> 3 (3)	2	1	NS
≤ 3 (27)	13	14	
Lymph node metastasis (n)			
No (12)	9	3	[*]0.0302
Yes (18)	6	12	
Distant metastasis (n)			
No (27)	13	14	NS
Yes (3)	2	1	
TNM Stage (n)			
I / II (27)	13	14	NS
III / IV (3)	2	1	
Recurrence (n)			
No (8)	6	2	NS
Yes (22)	9	13	

[a]Values are mean ± SD.
[b]NS, not significant.
[*]Student T-test P-value < 0.05.
